# Differential Response of Transcription Factors to Activated Kinases in Steroidogenic and Non-Steroidogenic Cells †

**DOI:** 10.3390/ijms232113153

**Published:** 2022-10-29

**Authors:** Kenley Joule Pierre, Jacques J. Tremblay

**Affiliations:** 1 Reproduction, Mother and Child Health, Room T3-67, CHU de Québec—Université Laval Research Centre, Québec, QC G1V 4G2, Canada; 2 Centre for Research in Reproduction, Development and Intergenerational Health, Department of Obstetrics, Gynecology and Reproduction, Faculty of Medicine, Université Laval, Québec, QC G1V 0A6, Canada

**Keywords:** Leydig cells, Steroidogenesis, signaling cascade, Nuclear receptors, bZIP factors, MEF2 factors, GATA4, CAMKI, PKA, ERK1/2

## Abstract

Hormone-induced Leydig cell steroidogenesis requires rapid changes in gene expression in response to various hormones, cytokines, and growth factors. These proteins act by binding to their receptors on the surface of Leydig cells leading to activation of multiple intracellular signaling cascades, downstream of which are several kinases, including protein kinase A (PKA), Ca^2+^/calmodulin-dependent protein kinase I (CAMKI), and extracellular signal-regulated protein kinase 1 and 2 (ERK1/2). These kinases participate in hormone-induced steroidogenesis by phosphorylating numerous proteins including transcription factors leading to increased steroidogenic gene expression. How these various kinases and transcription factors come together to appropriately induce steroidogenic gene expression in response to specific stimuli remains poorly understood. In the present work, we compared the effect of PKA, CAMKI and ERK1/2 on the transactivation potential of 15 transcription factors belonging to 5 distinct families on the activity of the *Star* gene promoter. We not only validated known cooperation between kinases and transcription factors, but we also identified novel cooperations that have not yet been before reported. Some transcription factors were found to respond to all three kinases, whereas others were only activated by one specific kinase. Differential responses were also observed within a family of transcription factors. The diverse response to kinases provides flexibility to ensure proper genomic response of steroidogenic cells to different stimuli.

## 1. Introduction

Leydig cells are located in the interstitial space between the seminiferous tubules in the mammalian testis where they produce two hormones, androgens (mainly testosterone) and insulin-like 3 (INSL3). During fetal life, these hormones are essential for masculinization on the male embryo while in postnatal life they are responsible for the development of internal and external male characteristics that occur at puberty, for the initiation and maintenance of spermatogenesis, and for bone metabolism. Testosterone synthesis requires several transporters and enzymes. The process begins with the shuttling of cholesterol, the substrate for all steroid hormones, from the outer to the inner mitochondrial membrane to deliver it to the CYP11A1 enzyme which makes pregnenolone. Pregnenolone then diffuses out of the mitochondria and reaches the smooth endoplasmic reticulum where it is transformed into testosterone by the sequential action of CYP17A1, HSD3B1, and HSD17B3 (reviewed in [1,2,3]). The transport of cholesterol, which constitutes the rate-limiting step in steroidogenesis, involves a large protein complex [4] which includes the steroidogenic acute regulatory (STAR) protein [5]. The importance of the STAR protein in Leydig cell steroidogenesis is supported by the existence of naturally occurring mutations in the human *Star* gene responsible for lipoid congenital adrenal hyperplasia and by inactivation of the *Star* gene in the mouse where males display female external genitalia, consistent with impaired testosterone production (reviewed in [6]).

Steroidogenesis in Leydig cells is regulated by several growth factors, cytokines, and hormones, the main one being the pituitary luteinizing hormone (LH). Binding of LH to its G protein-coupled receptor on the surface of Leydig cells activates adenylate cyclase leading to cAMP synthesis, which in turns activates several signaling pathways (reviewed in [1,7]). Similarly, growth factors and cytokines bind to their respective receptor leading to activation of intracellular signaling cascades (reviewed in [7,8,9,10,11]). Downstream of these pathways are several kinases, including protein kinase A (PKA), Ca^2+^/calmodulin-dependent protein kinase I (CAMKI), and extracellular signal-regulated protein kinase 1 and 2 (ERK1/2) (reviewed in [1]). These kinases participate in hormone-induced steroidogenesis by phosphorylating numerous proteins that include several transcription factors leading to increased steroidogenic gene expression (reviewed in [1,12]).

Because of the vital role of the STAR protein in steroidogenesis and because *Star* gene expression is strongly and rapidly induced in response to LH/cAMP stimulation (reviewed in [13]), the transcriptional regulation of the *Star* gene has been actively studied to identify *Star* promoter regulatory elements and their corresponding transcription factors that bind to these elements. Multiple transcription factors have been proposed to increase *Star* transcription (reviewed in [1,14,15]). These include members of the nuclear receptor family (SF1/NR5A1, LRH1/NR5A1, NUR77/NR4A1, COUP-TFII/NR2F2), bZIP family (cJUN, cFOS, CREB, CREM, C/EBPβ), GATA family (GATA4, GATA6), MADS box family (MEF2A, MEF2D), and the STAT domain family (STAT5B) (reviewed in [1,12,16,17,18,19,20,21]). While some of these transcription factors are de novo synthesized in response to LH/cAMP, such as NUR77 [22,23,24], most are activated by phosphorylation by one of three main kinases (PKA, CAMKI, ERK1/2). 

Despite the significant progress that have been made in identifying the signaling pathways and transcription factors regulating hormone-induced steroidogenesis in Leydig cells, important questions remain. For instance, in the majority of the studies performed so far, the effect of a given kinase on transcription factor activation and *Star* promoter activity has been limited to studying individual transcription factors. However, PKA, CAMKI, and ERK1/2 are all activated in response to LH/cAMP and therefore can target and activate several transcription factors simultaneously. In the present study, we have determined the ability of PKA, CAMKI and ERK1/2 to activate and functionally cooperate with 15 different transcription factors belonging to 5 distinct families. This allowed us to describe distinct transcription factor/kinase cooperation profiles that lead to increased *Star* gene transcription. 

## 2. Results

To determine whether the three main kinases (PKA, CAMKI, and ERK1/2), previously shown to activate *Star* transcription, can increase the transactivation potential of various transcription factors belonging to different families, we performed transient transfections in both a steroidogenic (MA-10 Leydig) and a non-steroidogenic (CV-1 fibroblast) cell line. Cells were cotransfected with a *Star* reporter plasmid along with expression vectors for the various transcription factors with or without expression vectors for the PKA catalytic subunit α, constitutively active CAMKI, and constitutively active MEK1 which phosphorylates and activates ERK1/2. 

### 2.1. Cell- and Kinase-Specific Cooperations with bZIP Family Members

**Figure 1:**
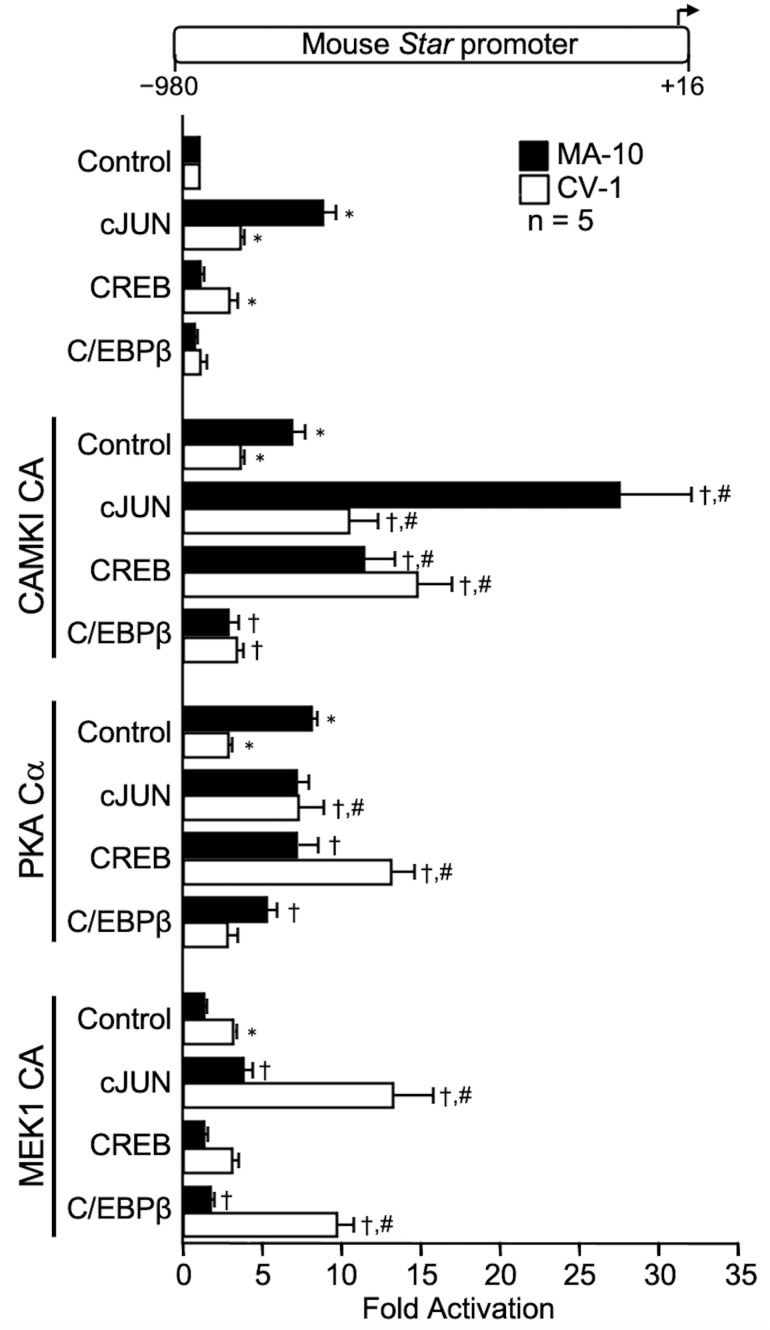
Transcriptional cooperation between bZIP family members and the kinases CAMKI constitutively active (CAMKI CA), PKA catalytic subunit alpha (PKA Cα), and MEK1 constitutively active (MEK1 CA activates ERK1/2) on the mouse *Star* promoter. MA-10 Leydig (black bars) and CV-1 fibroblast (white bars) cells were cotransfected with either an empty expression vector as a control or expression vectors for the different bZIP factors (cJUN, CREB, C/EBPaddress information is correct) and kinases (CAMKI CA, PKA Cα, MEK1 CA) individually or in combination as indicated, along with a −980 to +16 bp mouse *Star* reporter. Results are shown as Fold Activation over control ± SEM. An asterisk (*) represents a statistically significant difference in activation by the transcription factor or the kinase compared to control (empty expression vector, value set at 1, *p* < 0.05). A dagger (†) represents a statistically significant difference in activation between the transcription factor + the kinase compared to the corresponding transcription factor alone (*p* < 0.05). A hashtag (#) represents a statistically significant difference in activation between the transcription factor + the kinase compared to the kinase alone (*p* < 0.05). The number (n) of replicates is indicated.

We first tested three members of the bZIP family (cJUN, CREB, and C/EBPβ) alone. As shown in Figure 1, CREB and cJUN activated the *Star* promoter in both MA-10 Leydig and CV-1 fibroblast cells, with the activation by cJUN being stronger in MA-10 cells (~10-fold vs. ~4-fold in CV-1 cells). In contrast, CREB-mediated activation was stronger in CV-1 cells (~4-fold). There was a tendency towards an activation by C/EBPβ but it did not reach statistical significance (Figure 1). We next repeated these experiments but in the presence of a kinase. All three kinases (CAMKI CA, PKA Cα, MEK1 CA) increased *Star* promoter activity (up to 7-fold in MA-10 cells and up to 3.8-fold in CV-1 cells) on their own. The activation by CAMKI CA and PKA Cα was much stronger in MA-10 Leydig cells than in CV-1 fibroblast cells, while the reverse was true for MEK1 CA. This indicated that the kinases activate transcription factors already present in the cells, mainly in MA-10 cells for CAMKI CA and PKA Cα, and in CV-1 cells for MEK1 CA. When assessed for functional cooperation, CAMKI CA potently enhanced cJUN- (up to 25-fold) and CREB- (up to 10-fold) dependent activation of the *Star* promoter in both MA-10 Leydig and CV-1 fibroblast cells (Figure 1). PKA Cα also cooperated with cJUN and with CREB but only in CV-1 cells (Figure 1). Finally, a cooperation between MEK1 CA and cJUN (up to 15-fold) and C/EBPβ (up to 10-fold) was observed solely in CV-1 fibroblasts (Figure 1). Together, these results indicated that although the transactivation by all three bZIP family members is enhanced in the presence of a kinase, the resulting cooperation is kinase- and cell type-specific, with cJUN and CREB activity being mainly enhanced by CAMKI CA and PKA Cα, and MEK1 CA (ERK1/2) with C/EBPβ and cJUN.

### 2.2. Some Nuclear Receptors Cooperate with All Three Kinases while Others Are Stimulated by a Single Kinase

**Figure 2:**
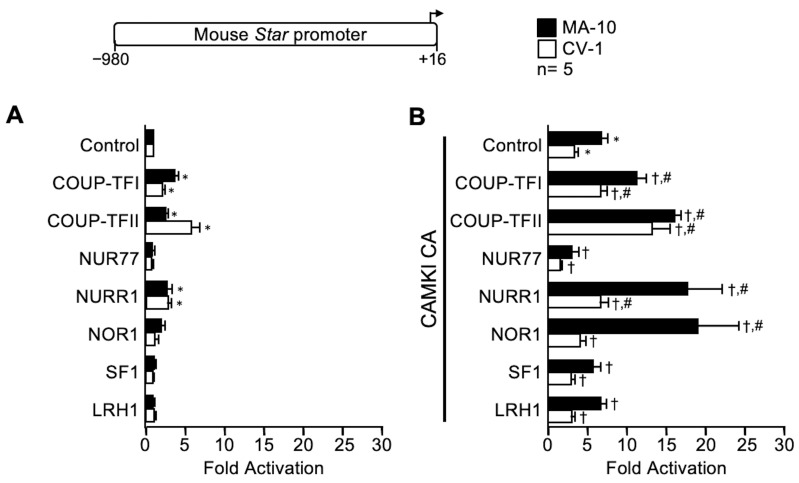

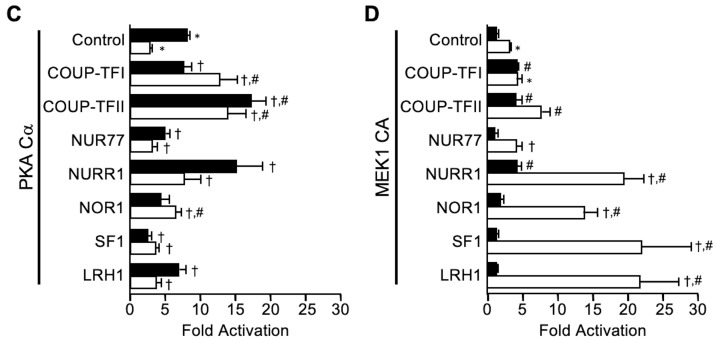
Specific cooperation between select members of the nuclear receptor family and the kinase CAMKI constitutively active (CAMKI CA), PKA catalytic subunit alpha (PKA Cα), and MEK1 constitutively active (MEK1 CA activates ERK1/2) on the mouse *Star* promoter. MA-10 Leydig (black bars) and CV-1 fibroblast (white bars) cells were cotransfected with a −980 to +16 bp mouse *Star* reporter along with an empty expression vector as a control or expression vectors for the different nuclear receptors (COUP-TFI, COUP-TFII, NUR77, NURR1, NOR1, SF1, LRH1) used individually (**A**) or in combination with CAMKI CA (**B**), PKA Cα (**C**), and MEK1 CA (**D**). Results are shown as Fold Activation over control ± SEM. An asterisk (*) represents a statistically significant difference in activation by the transcription factor or the kinase compared to the control empty expression vector (whose value was set at 1, *p* < 0.05). A dagger (†) represents a statistically significant difference in activation between the transcription factor + the kinase compared to the corresponding transcription factor alone (*p* < 0.05). A hashtag (#) represents a statistically significant difference in activation between the transcription factor + the kinase compared to the kinase alone (*p* < 0.05). The number of replicates is indicated.

The nuclear receptor family is composed of 48 members, several of which are expressed in Leydig cells where they regulate expression of numerous genes, including *Star* (reviewed in [16]). As previously demonstrated [23,25], members of the NR2F (COUP-TFI, COUP-TFII) and NR4A families activated the *Star* promoter both in MA-10 and CV-1 cells (Figure 2). We next determined whether cooperation, between the kinases and seven nuclear receptors belonging to three distinct families, occurs on the *Star* promoter. As shown in Figure 2, the activation of the *Star* promoter by COUP-TFI and COUP-TFII (NR2F family members) was significantly enhanced by CAMKI CA and PKA Cα in both MA-10 and CV-1 cells. On the other hand, all three kinases activated at least one of the NR4A family members (CAMKI CA with NURR1 and NOR1; PKA Cα with NOR1, and MEK1 CA with NURR1 and NOR1) in MA-10 Leydig and/or CV-1 fibroblast cells. Finally, the two members of the NR5A family, SF1 and LRH1, were only stimulated by MEK1 CA and exclusively in CV-1 fibroblast cells (Figure 2). Together these results establish that stimulation of nuclear receptor activity is highly kinase-dependent, which is determined by the family the nuclear receptor belongs to.

### 2.3. MEF2 Factors Cooperate with PKA Cα and MEK1 CA in CV-1 Cells

**Figure 3:**
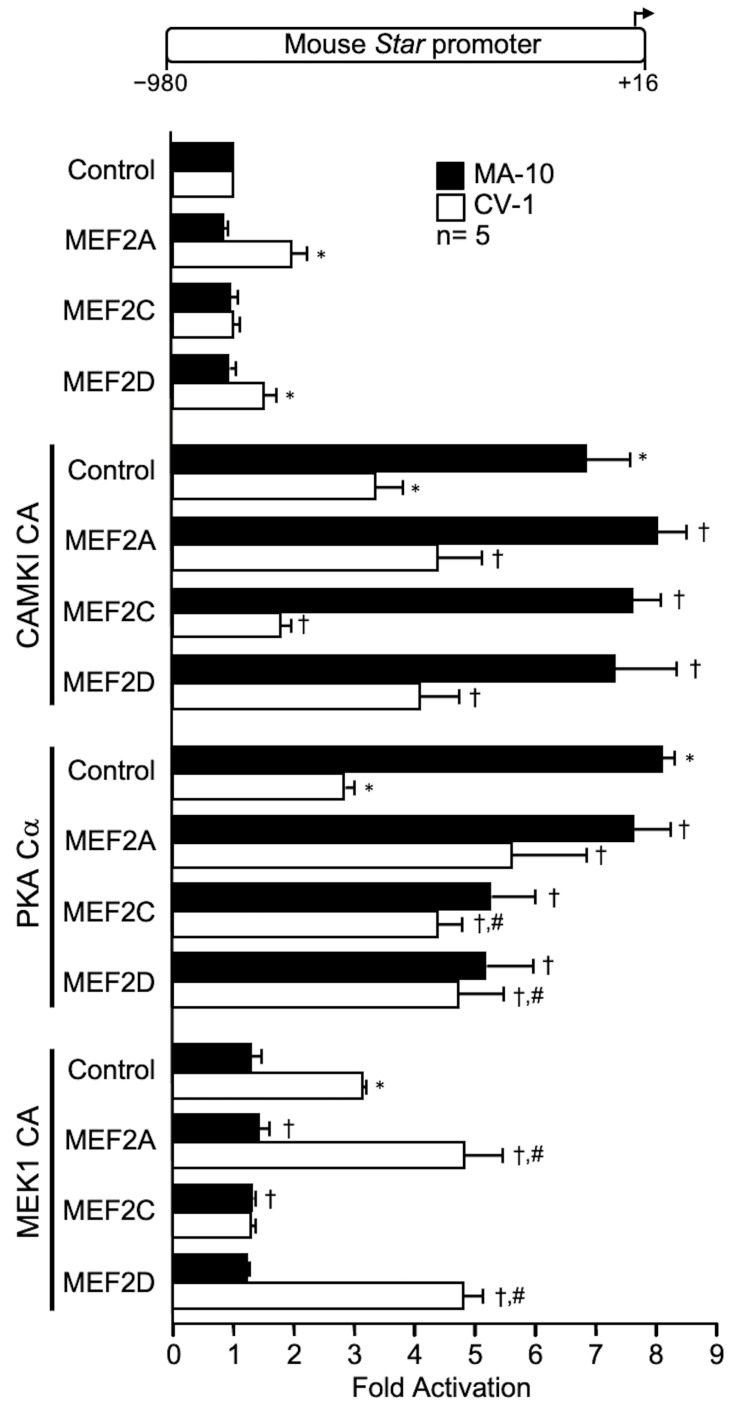
Cooperation between members of MADS box family of transcription factors and the kinases PKA catalytic subunit alpha (PKA Cα) and MEK1 constitutively active (MEK1 CA activates ERK1/2) on the mouse *Star* promoter. MA-10 Leydig (black bars) and CV-1 fibroblast (white bars) cells were cotransfected with an empty expression vector as a control or expression vectors for various MADS box family members (MEF2A, MEF2C, MEF2D) and kinases (CAMKI CA, PKA Cα, MEK1 CA) individually or in combination as indicated, along with a −980 to +16 bp mouse *Star* reporter. Results are shown as Fold Activation over control ± SEM. An asterisk (*) represents a statistically significant difference in activation by the transcription factor or the kinase compared to control (empty expression vector, value set at 1, *p* < 0.05). A dagger (†) represents a statistically significant difference in activation between the transcription factor + the kinase compared to the corresponding transcription factor alone (*p* < 0.05). A hashtag (#) represents a statistically significant difference in activation between the transcription factor + the kinase compared to the kinase alone (*p* < 0.05). The number (n) of replicates is indicated.

Of the four MEF2 family members, three are expressed in Leydig cells (MEF2A, 2C and 2D) where they are known to regulate the expression of several genes [26,27,28,29,30,31]. To determine whether the three kinases could enhance MEF2 transactivation potential, transient transfections were performed in MA-10 Leydig and CV-1 fibroblast cells. As shown in Figure 3, cooperation was observed between PKA Cα and MEF2C and MEF2D as well as between MEK1 CA and MEF2A and MEF2D. These cooperations were observed exclusively in CV-1 cells, which is consistent with the fact that MA-10 Leydig cells already contain high levels of MEF2 proteins [28]. 

### 2.4. GATA4 Activity Is Strongly Enhanced in the Presence of CAMKI CA, PKA Cα, and MEK1 CA 

**Figure 4:**
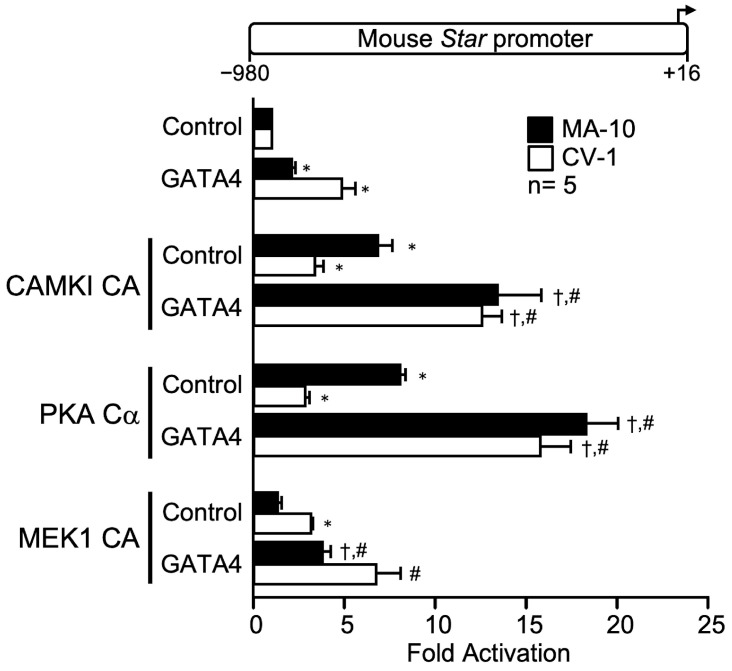
The zinc finger transcription factor GATA4 cooperates with CAMKI constitutively active (CAMKI CA), PKA catalytic subunit alpha (PKA Cα), and MEK1 constitutively active (MEK1 CA activates ERK1/2) on the mouse *Star* promoter. MA-10 Leydig (black bars) and CV-1 fibroblast (white bars) cells were cotransfected with a control empty expression vector or expression vectors for GATA4 and the different kinases (CAMKI CA, PKA Cα, MEK1 CA) individually or in combination as indicated, along with a −980 to +16 bp mouse *Star* reporter. Results are shown as Fold Activation over control ± SEM. An asterisk (*) represents a statistically significant difference in activation by the transcription factor or the kinase compared to control (empty expression vector, value set at 1, *p* < 0.05). A dagger (†) represents a statistically significant difference in activation between the transcription factor + the kinase compared to the corresponding transcription factor alone (*p* < 0.05). A hashtag (#) represents a statistically significant difference in activation between the transcription factor + the kinase compared to the kinase alone (*p* < 0.05). The number (n) of replicates is indicated.

The GATA4 transcription factor is a known activator of *Star* promoter activity in Leydig cells [27,32,33,34,35,36,37]. Consistent with this, GATA4 was found to activate the *Star* promoter both in MA-10 Leydig (3-fold) and CV-1 fibroblast (5-fold) cells (Figure 4). Combination of GATA4 with CAMKI CA (up to 13-fold), and PKA Cα (up to 16-fold), enhanced GATA4 activation both in MA-10 and CV-1 cells (Figure 4). A MEK1 CA/GATA4 cooperation was only observed in MA-10 Leydig cells (Figure 4). These results suggest that GATA4 is a common target downstream of diverse intracellular signaling pathways and kinases.

### 2.5. STAT5B Cooperates with All Kinases 

**Figure 5:**
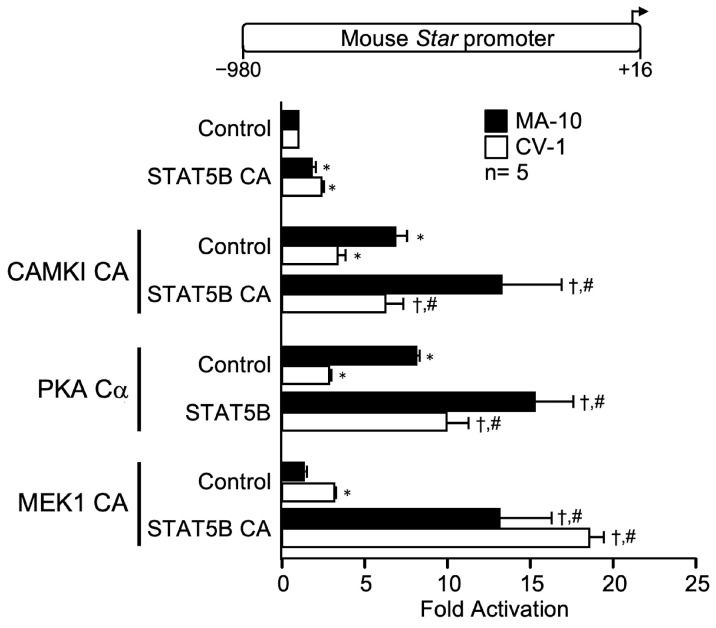
Cooperation between STAT5B and CAMKI constitutively active (CAMKI CA), PKA catalytic subunit alpha (PKA Cα), and MEK1 constitutively active (MEK1 CA activates ERK1/2) on the mouse *Star* promoter. MA-10 Leydig (black bars) and CV-1 fibroblast (white bars) cells were cotransfected with an empty expression vector as a control or expression vectors for constitutively active STAT5B (STAT5B CA) and the different kinases (CAMKI CA, PKA Cα, MEK1 CA) individually or in combination as indicated, along with a −980 to +16 bp mouse *Star* reporter. Results are shown as Fold Activation over control ± SEM. An asterisk (*) represents a statistically significant difference in activation by the transcription factor or the kinase compared to control (empty expression vector, value set at 1, *p* < 0.05). A dagger (†) represents a statistically significant difference in activation between the transcription factor + the kinase compared to the corresponding transcription factor alone (*p* < 0.05). A hashtag (#) represents a statistically significant difference in activation between the transcription factor + the kinase compared to the kinase alone (*p* < 0.05). The number (n) of replicates is indicated.

STAT5B is a transcription factor activated in response to growth hormone (reviewed in [38]). Recently, STAT5B was found to mediate GH-induced *Star* gene expression in Leydig cells [39]. In agreement with this, a constitutively active form of STAT5B (STAT5B CA) activated the *Star* promoter by ~3-fold in MA-10 Leydig and CV-1 fibroblast cells (Figure 5). Combination of STAT5B CA with any kinase (CAMKI CA, PKA Cα, MEK1 CA) resulted in a synergistic activation of the *Star* promoter reaching nearly 20-fold in both MA-10 Leydig and CV-1 fibroblast cells (Figure 5). This indicates that STAT5B is a versatile transcription factor that can be stimulated by various kinases.

## 3. Discussion

**Figure 6:**
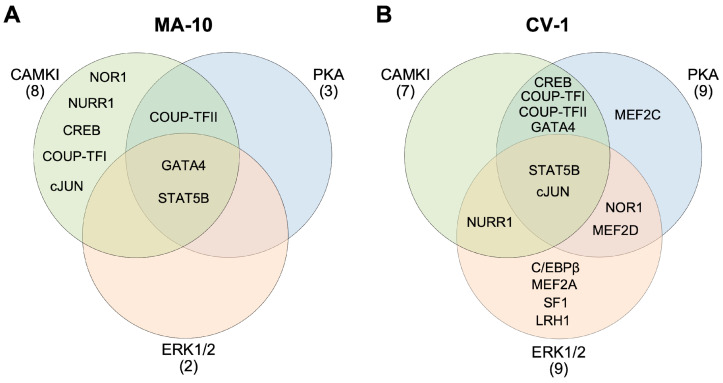
Cooperation of transcription factors and kinases on the *Star* promoter in steroidogenic (MA-10) vs. non-steroidogenic (CV-1) cells. The three-set Venn diagram highlights the transcription factors that cooperate with CAMKI CA (green circles), PKA Cα (blue circles), and MEK1 CA (activating ERK1/2, orange circles) in MA-10 Leydig (**A**) and in CV-1 fibroblast (**B**) cells.

Hormone-induced Leydig cell steroidogenesis is a strictly regulated process that requires changes in gene expression and protein phosphorylation. Expression of the *Star* gene, which is induced upon stimulation of steroidogenesis has been the subject of intense studies since its discovery nearly 30 years ago [5]. This has led to the identification of several transcription factors that act via regulatory motifs clustered within the proximal *Star* promoter (reviewed in [1,40,41]). Some of these transcription factors are activated by various kinases, including PKA, CAMKI and ERK1/2, that are themselves activated in response to different hormones known to increase *Star* expression and steroidogenesis in Leydig cells (reviewed in [1]). In other systems, some of these transcription factors are known to be activated downstream of different signaling pathways and kinases. How these kinases and transcription factors are integrated to appropriately induce *Star* transcription in response to specific stimuli remains poorly understood. The main objective of this work was to compare the effect of three key kinases on the transactivation potential of 15 transcription factors belonging to 5 distinct families on *Star* promoter activity. As described below, this allowed us to validate existing cooperations as well as to identify new ones. As summarized in Figure 6, some transcription factors were activated by multiple kinases indicating that they may act downstream of multiple signaling cascades. Furthermore, the fact that all the transcription factors and kinases were assessed simultaneously allowed for a direct comparison of the significance (or strength) of the transcription factor/kinase cooperation (summarized in Table 1).

Experiments were performed in two cell lines, the MA-10 Leydig cell line and the CV-1 fibroblast cell line. MA-10 Leydig cells endogenously express the various transcription factors and kinases tested as well as the *Star* gene. Therefore, expression of a kinase often leads to a significant activation since it activates transcription factors already present in the cells. The transcription factor could be the factor of interest or another transcription factor. On the other hand, CV-1 cells are considered heterologous cells since they do not express the *Star* gene and all transcription factors normally found in a Leydig cell. Using a heterologous cell line therefore allows to detect promoter activation by a transcription factor or a cooperation between factors that would otherwise be undetectable in a homologous cell line like MA-10 Leydig cells. In agreement with this, the activity of some transcription factors was only stimulated by a kinase in CV-1 cells (Figure 6).

### 3.1. New Cooperations between Transcription Factors and Kinases

The roles of PKA and MEK1 (activating ERK1/2) have been well characterized and they are known to stimulate various transcription factors in different cell types. However, their effects on most of the transcription factors in the activation of the *Star* promoter remained to be characterized. 

#### 3.1.1. PKA-Induced Transcription Factor Activity

Of the 15 transcription factors tested, 3 were activated by PKA Cα in MA-10 Leydig cells while in CV-1 fibroblast cells, the same 3 plus an additional 6 (9 in total) were activated (blue circles in Figure 6). PKA is known to stimulate cJUN activity, but this was shown using a synthetic reporter [42]. We found that cJUN-dependent activation of the *Star* promoter is significantly induced by PKA Cα in CV-1 cells. Similarly, CREB is a classic target of PKA [43] and therefore the cooperation between CREB and PKA Cα on the *Star* promoter was not unexpected. We also found that PKA Cα stimulated the activity of the orphan nuclear receptors COUP-TFI and COUP-TFII on the *Star* promoter in both MA-10 and CV-1 cells, which was unknown for the *Star* gene. A previous study in a different system did report that PKA could cooperate with COUP-TFI on the *Vitronectin* promoter [44]. Similarly, the stimulatory effect of PKA Cα on NR4A family members (NUR77, NURR1, NOR1) has only been observed on the *Pomc* promoter in pituitary corticotrope cells [45]. Here, we found that PKA Cα enhanced NOR1-dependent activation of the *Star* promoter. Of the different MEF2 family members, we found that PKA Cα increased the transactivation potential of MEF2C and MEF2D on the *Star* promoter. A previous study in the heart found that PKA represses MEF2A activity [46], indicating the existence of cell type-specific responses. The transcription factor GATA4 has been described as a direct target for PKA, which increases its transactivation potential on the *Star* promoter [47]. Our current study reproduces this observation, thus validating the appropriateness of our experimental system. We also observed a potent stimulation of STAT5B-dependent activation of the *Star* promoter by PKA Cα both in MA-10 Leydig and CV-1 fibroblast cells. STAT5B is known to be phosphorylated by members of the JAK kinase family in response to growth hormone (reviewed in [48]) in many cell types, including in Leydig cells [49]. Whether PKA directly phosphorylates STAT5B or whether it acts on STAT5B-interacting partner remains to be established. 

#### 3.1.2. MEK1-Induced Transcription Factor Activity

We found MEK1 CA to enhance the transactivation potential of 2 transcription factors in MA-10 Leydig cells and 9 in CV-1 fibroblast cells (orange circles in Figure 6). The ERK1/2 kinases activated by MEK1 are known to phosphorylate and activate GATA4-dependent transactivation in the heart [50] as well in the mouse testis where phosphorylation of GATA4 Ser105 is required for testosterone production [51]. However, a direct effect of MEK1 CA on GATA4-dependent activation of the *Star* promoter as we observed had never been reported. This suggests that in addition to being stimulated by PKA, GATA4 activity is also activated by ERK1/2 on the *Star* promoter. Similar to GATA4, the transactivation potential of STAT5B on the *Star* promoter was also enhanced in the presence of MEK1 CA. A cooperation between STAT5B and ERK1/2 has never been reported. We also found that MEK1 CA enhances the activity of the NR5A nuclear receptors SF1 and LRH1. Although ERK1/2-mediated SF1 and LRH1 phosphorylation can stimulate their transactivation potential [52,53] in HeLa and JEG-3 cells on either the *Cyp7a1* or a synthetic promoter, this potentiation had not been reported on the *Star* promoter. With respect to the NR4A family of nuclear receptors, ERK1/2 was previously reported to phosphorylate and stimulate NUR77 leading to enhanced activity of an artificial promoter in AtT-20 corticotrope cells [54]. Although we did not observe any effect of MEK1 CA (ERK1/2) on NUR77-dependent activation of the *Star* promoter, MEK1 CA did nonetheless significantly enhance the activity of the other two NR4A family members NURR1 and NOR1. This suggests the existence of a cell- and promoter-dependent response of transcription factors to different kinases. The bZIP factors cJUN and C/EBPβ were also found to cooperate with MEK1 CA on the *Star* promoter. This was unexpected since activated ERK1/2 is believed to inactivate cJUN, while other MAPK members such as JNKs phosphorylate and activate cJUN (reviewed in [55]). In 3T3-L1 preadipocytes, C/EBPβ is phosphorylated in a MEK1-dependent manner, stimulating its transactivation potential [56,57], which is similar to what we observed on the *Star* promoter. 

#### 3.1.3. CAMKI-Induced Transcription Factor Activity

CAMKI is the most recently identified kinase in Leydig cells and consequently the least studied in these cells. The transactivation potential of 8 transcription factors in MA-10 cells and 7 in CV-1 cells was enhanced by CAMKI CA (green circles in Figure 6). Similar to what we observed in the present study, the transactivation potential of all members of the NR4A family of nuclear receptors (NUR77, NURR1 and NOR1) and of SF1 (NR5A1) was previously shown to be increased in the presence of CAMKI CA on the *Star* promoter [23]. The nuclear receptors COUP-TFI and COUP-TFII were both activated in the presence of CAMKI CA, as revealed by the synergistic activation of the *Star* promoter in both MA-10 and CV-1 cells. The two COUP-TF factors have not been reported to be stimulated by CAMKI, although a study showed that the activity of COUP-TFI is potentiated by the related CAMKIV in neuronal cells [58]. Our study also revealed that CAMKI CA enhances the activity of cJUN on the *Star* promoter both in MA-10 and in CV-1 cells, similar what was recently reported on the *Cx43* promoter in MA-10 Leydig cells [59]. We observed a strong stimulation of the activity of CREB by CAMKI CA on the *Star* promoter. CREB is known to be phosphorylated in different cell types by CAMKI and CAMKIV leading to an increase in its transactivation potential [60,61]. We found that CAMKI CA significantly enhanced the transactivation potential of GATA4 and STAT5B on the *Star* promoter in both MA-10 and CV-1 cells. Both transcription factors were not previously known to cooperate with CAMKI. 

### 3.2. Versatility in Transcription Factor Response to Different Kinases

An interesting observation in our findings is the fact that the transactivation potential of most transcription factors was stimulated by more than one kinase. This is clearly apparent in the Venn diagrams presented in Figure 6. Some transcription factors were found to respond to all three kinases such as GATA4 and STAT5B in MA-10 Leydig cells, and cJUN and STAT5B in CV-1 cells. This flexibility in how a given transcription factor responds to different kinases suggests that the transcription factor can likely mediate the effects of different stimuli thus ensuring proper genomic response. 

Another form of versatility exists within a family of transcription factors where different members respond to different kinases. For instance, in the nuclear receptor family, NR4A members (NUR77, NURR1, NOR1) responded to all three kinases (PKA Cα, CAMKI CA, MEK1 CA), NRF2 members (COUP-TFI, COUP-TFII) were stimulated by two kinases (PKA Cα, CAMKI), and NR5A members (SF1, LRH1) were only activated by MEK1 CA. Since most of these nuclear receptors can bind to the same response element in a promoter, this differential response to a kinase might provide the specificity needed to ensure the proper nuclear receptor is activated downstream of a signaling cascade leading to increased gene expression.

In conclusion, our current work has identified several transcription factors whose transactivation potential is stimulated by different kinases. Some of these transcription factors were previously reported to be directly phosphorylated by the kinase. However, for several others identified in our current work, it remains to be determined whether they are a direct target of the kinase. It is possible that the kinase might instead phosphorylate another protein that can then interact with the transcription factor leading to a cooperation between the two transcription factors. In this case, kinase-mediated phosphorylation of a factor might render it more receptive to interactions and cooperations with other transcription factors. This could be tested by determining whether the different kinases further enhance known cooperations between two transcription factors. Additional work is needed to answer these questions.

## 4. Materials and Methods

### 4.1. Plasmids

The mouse *Star* luciferase promoter construct (−980/+16 bp) was previously described [34]. The MEF2A and MEF2D [27,28,30] and GATA4 [62] expression plasmids were previously described. The mouse MEF2C expression plasmid was generated by amplifying the complete coding sequence by PCR (forward primer 5′-CCC AAG CTT ATG GGG AGA AAA AAG ATT CAG ATT-3′, reverse primer 5′-GCT CTA GAT CAT GTT GCC CAT CCT TCA-3′) and subcloning the resulting PCR product into the HindIII and XbaI cloning sites of the pcDNA3.1 expression vector (Invitrogen Canada, Burlington, ON, Canada). The following expression plasmids were sourced from different research groups: rat NUR77/NR4A1, NURRI/NURR1, NOR1/NR4A3 [63], mouse SF1/NR5A1 [64], human LRH1/NR5A2 [65], cJUN [66], CREB and PKA catalytic subunit α [67], constitutively active MEK1 [68], C/EBPβ [69], mouse COUP-TFI/NR2F1 and COUP-TFII/NR2F2 [70], constitutively active STAT5B [71], and constitutively active CAMKI [72].

### 4.2. Cells Culture, Transfections, and Reporter Assays

Mouse MA-10 Leydig cells (ATCC, Manassas, VA, USA, Cat# CRL-3050, RRID:CVCL_D789) were grown in DMEM/F12 medium supplemented with 2.438 g/L sodium bicarbonate, 3.57 g/L HEPES, and 15% horse serum on gelatin-coated plates. African green monkey kidney fibroblast CV-1 cells (ATCC, Cat# CRL-6305, RRID:CVCL_0229) were grown in DMEM medium supplemented with 3.7 g/L HEPES, and 10% newborn calf serum. Penicillin and streptomycin sulphate were added to the cell culture media to a final concentration of 50 mg/L, and all cell lines were kept at 37 °C, 5% CO_2_ in a humidified incubator. All cell lines were validated by morphology and Leydig cell lines by quantifying steroidogenic output (progesterone for MA-10) as previously described [25,27,39,73,74,75,76,77]. MA-10 (60,000 cells per well) and CV-1 (25,000 cells per well) were transiently transfected using polyethylenimine hydrochloride (PEI) (Sigma-Aldrich Canada, Oakville, ON, Canada) as previously described [39,78] or the calcium phosphate co-precipitation method as described in [23,24]. Briefly, the cells were seeded in 24-well plates and cotransfected with 400 ng of the mouse *Star* −980/+16 bp reporter vector along with 100 ng of an empty expression vector (pcDNA3.1 as control), or expression vectors for the various transcription factors (50 ng) or kinases (30 ng) individually (completed to 100 ng with the empty pcDNA3.1 expression vector to keep the total amount of expression vector to 100 ng), or the combination of a transcription factor (50 ng) plus a kinase (30 ng) and empty pcDNA3.1 (20 ng). For the calcium phosphate co-precipitation method, 1 µg of SP64 inert plasmid was also added as carrier. Sixteen hours post transfection, the media was replaced, and the cells were grown for additional 32 h. Cells were then lysed, the lysates were collected, and the luciferase measurements was performed using a Tecan Spark 10M multimode plate reader (Tecan, Morrisville, NC, USA) as previously described [39,78]. The number of experiments, each performed in triplicate, is indicated in each figure. All the cDNAs are cloned in an expression plasmid containing a strong promoter (CMV), which leads to high expression levels. The quantity of expression vector needed to obtain specific and optimal promoter activation was determined by performing a dose–response curve as described in [25]. Western blots were routinely performed to ensure overexpression was achieved, especially when using a new DNA plasmid preparation.

### 4.3. Statistical Analysis

Comparisons between two groups were performed using an unpaired Student *t*-test (GraphPad Prism, GraphPad Software, San Diego, CA, USA, version 9.4.1 (458)). For all statistical analyses, *p* < 0.05 was considered significant.

## Figures and Tables

**Table 1:** Transactivation potential of transcription factors by different kinases.

		Control		CAMKI CA		PKA Cα		MEK1 CA	
		MA-10	CV-1	MA-10	CV-1	MA-10	CV-1	MA-10	CV-1
	Control	1.0	1.0	6.9	3.4	8.1	2.8	1.3	3.1
bZIP	cJUN	8.8	3.5	27.5	10.4	7.2	7.2	3.8	13.2
	CREB	1.0	2.9	11.4	14.7	7.1	13.1	1.3	3.1
	C/EBPβ	0.7	1.1	2.8	3.4	5.1	2.8	1.7	9.7
Nuclear receptors	COUP-TFI	3.7	2.1	11.4	6.7	7.6	12.8	4.1	4.2
	COUP-TFII	2.5	5.8	16.0	13.4	17.2	13.9	4.0	7.6
	NUR77	0.8	0.7	3.1	1.6	5.0	3.1	1.0	4.1
	NURR1	2.6	2.8	17.9	6.7	15.1	7.7	4.1	19.3
	NOR1	2.0	1.1	19.2	4.1	4.3	6.5	1.8	13.7
	SF1	1.0	0.9	5.8	2.9	2.5	3.6	1.3	21.8
	LRH1	0.9	1.1	6.7	3.2	6.9	3.7	1.3	21.5
MADS box	MEF2A	0.8	2.0	8.0	4.4	7.6	5.6	1.4	4.8
	MEF2C	1.0	1.0	7.6	1.8	5.3	4.4	1.3	1.3
	MEF2D	0.9	1.5	7.3	4.1	5.2	4.7	1.2	4.8
GATA	GATA4	2.1	4.9	13.5	12.6	18.4	15.9	3.8	6.8
STAT	STAT5B	1.8	2.4	13.3	6.3	15.3	10.0	13.1	18.6

Fold activations are compared to the control (empty expression vector) for which the value was set to 1.

## Data Availability

All data generated or analyzed during this study are included in this article.
